# Exploratory Cross-Trial Non-Inferiority Analysis of 2% Versus 1% Sodium Hyaluronate in Knee Osteoarthritis

**DOI:** 10.3390/jcm15176571

**Published:** 2026-08-26

**Authors:** Fernando Costa, Pilar Coronel Granado, Laura Casas, Diego Maisonave Berdasco, Débora Renz Barreto Vianna

**Affiliations:** 1 Corporate Clinical Studies Department, Adium Pharma, Ciudad Autónoma de Buenos Aires B1603APO, Argentina; debora.vianna@adium.com.br; 2 Scientific Department, Meiji Pharma Spain, Alcalá de Henares, 28802 Madrid, Spain; 3 CR-Biostatistics, Evidenze Clinical Research, 28109 Madrid, Spain; 4 Scientific Advisory and Medical Writing Department, Evidenze Clinical Research, 28109 Madrid, Spain

**Keywords:** osteoarthritis, knee osteoarthritis, viscosupplementation, sodium hyaluronate, non-inferiority

## Abstract

**Objectives**: Our primary objective was to compare the 12-month efficacy of two dosing regimens of intra-articular sodium hyaluronate (SH) in patients with knee osteoarthritis (OA) using a cross-study analysis within a non-inferiority framework. **Methods**: This study combined individual patient data from two prospective multicenter trials with similar outcome measures: the Trueba Davalillo study (five injections of 1% SH) and the SOYA Trial (single injection of 2% SH, with the possibility of retreatment after 6 months). The primary endpoint of this cross-study analysis was the relative change in pain intensity on a 100 mm visual analog scale (VAS) from baseline to 12 months. Secondary outcomes included changes in the Western Ontario and McMaster Universities Osteoarthritis Index (WOMAC) and the proportion of patients achieving the Minimal Clinically Important Improvement (MCII). Non-inferiority was defined for the primary endpoint as the lower bound of the 95% confidence interval for the between-group difference remaining above −8%. **Results**: The modified intention-to-treat (mITT) population included 97 patients in the 1% SH group and 98 in the 2% SH group. At 12 months, the mean relative reduction in pain intensity was 33.61% for 1% SH and 37.67% for 2% SH (mean difference: 4.06%; 95% CI: −6.37% to 14.50%), compatible with non-inferiority (per the predefined −8% criterion) in this exploratory cross-trial analysis. No significant between-group differences were found in adjusted analyses for pain or WOMAC scores at 12 months. At 12 months, MCII responder rates were higher in the 1% SH group. **Conclusions**: This exploratory cross-trial analysis found results compatible with non-inferiority of 2% SH relative to 1% SH in improving pain and physical function over 12 months in patients with knee OA.

## 1. Introduction

Osteoarthritis (OA) is a chronic, progressive joint disease characterized by cartilage degradation, synovial inflammation, subchondral bone remodeling, and pain [1]. Globally, OA is estimated to affect more than 500 million people, including 23% of the population aged ≥40 years [2,3]. Pain associated with OA significantly limits physical activity and is a major contributor to global disability, accounting for approximately 2.4% of all years lived with disability worldwide [2,3]. In the United States, OA reduces work capacity in two-thirds of affected individuals and contributes to increased absenteeism in over 20% of cases [3]. The knee is the most affected site, accounting for an estimated 60% to 85% of OA diagnoses [4,5,6].

Despite being a highly prevalent and disabling condition, the pathophysiology of knee OA remains incompletely understood, and no disease-modifying treatment is currently available [7,8]. Management still relies on combining nonpharmacological and pharmacological strategies to relieve symptoms and improve function [9,10]. Common pharmacologic options, such as acetaminophen, nonsteroidal anti-inflammatory drugs, and intra-articular corticosteroids, are limited by lack of response and systemic adverse effects, particularly in older adults with comorbidities [11,12,13,14]. Intra-articular hyaluronic acid has emerged as a safer local alternative, especially in patients who cannot tolerate or do not respond to first-line therapies [15,16]. Hyaluronic acid improves the viscoelastic properties of synovial fluid, thereby enhancing joint lubrication and reducing mechanical stress and inflammation [17]. Although both 1% and 2% sodium hyaluronate (SH) formulations are used in clinical practice, they differ not only in concentration but also in treatment regimen, with the former typically administered as a series of weekly injections and the latter as a single injection with optional retreatment. In the absence of direct head-to-head studies, it remains uncertain whether the potential practical advantages of the single-injection approach are achieved without compromising long-term symptom relief [18]. Therefore, comparing the effectiveness of these two formulations represents a clinically relevant question for treatment selection.

Two open-label, prospective, multicenter studies independently evaluated the safety and efficacy of intra-articular injections of 1% and 2% SH in patients with knee OA over a 12-month period [19,20]. These studies used comparable outcome measures and follow-up schedules, allowing for indirect comparisons between the two formulations. Leveraging these datasets, we conducted an exploratory cross-trial analysis applying a non-inferiority statistical framework to compare the efficacy of 1% versus 2% SH in improving pain outcomes in OA over one year of follow-up. This study does not constitute a head-to-head randomized comparison; rather, it represents an indirect, exploratory comparison between two trials conducted independently, a distinction that should inform the interpretation of the findings reported herein.

## 2. Materials and Methods

### 2.1. Data Sources

This non-inferiority analysis was based on individual patient data from two prospective clinical trials evaluating intra-articular SH for knee OA: the Trueba Davalillo [16] study and the SOYA Trial [20]. A detailed comparison of the methodological differences between the two trials is provided in Supplementary Table S1.

This study followed relevant principles of the Consolidated Standards of Reporting Trials (CONSORT) extension for reporting noninferiority and equivalence trials [21], adapted as applicable to a cross-trial, individual patient-data analysis. Unlike a randomized head-to-head non-inferiority trial, patients were not randomized between the 1% and 2% SH treatment groups; instead, this analysis combines data from two independently conducted trials with distinct designs (one randomized and controlled, the other uncontrolled), as detailed in Section 2.1.1 and Section 2.1.2 and discussed as a key limitation in Section 4.

#### 2.1.1. The Trueba Davalillo Study

The Trueba Davalillo study [19] was a randomized, parallel-group trial conducted in Mexico. The study compared two treatment strategies in patients with knee OA: a regimen of five intra-articular injections of 1% SH (Suprahyal^®^, Meiji Pharma Spain, S.A., Madrid, Spain), whose efficacy, safety and carry-over effect with repeated cycles have been previously reported [22]. The treatment was administered once a week for five consecutive weeks, versus two intra-articular injections of betamethasone given four weeks apart. Follow-up assessments were conducted at 3, 6, 9, and 12 months after the first injection.

Eligible participants were men or women aged 40 to 85 years with a diagnosis of knee OA confirmed by radiographic evidence of grade II or III OA according to the Kellgren–Lawrence scale. Inclusion required a body mass index (BMI) < 35 kg/m^2^ and the absence of inflammatory arthritis, previous knee surgery, significant joint deformities, or metabolic conditions such as diabetes mellitus or metabolic syndrome.

For the SH group, the trial enrolled 97 patients in the modified intention-to-treat (mITT; patients with at least one efficacy assessment after randomization) population, and by the end of the study, 89 patients completed the 12-month follow-up without major protocol deviations (per-protocol [PP] population). The betamethasone group included 98 patients in the mITT population and 91 in the PP population.

#### 2.1.2. The SOYA Trial

The SOYA Trial [20] was a phase III, prospective, multicenter, uncontrolled clinical study conducted in Spain. The trial assessed the efficacy, safety, and duration of effect of a single intra-articular injection of 2% SH (Adant Plus^®^ [identified as MPS-HA2% at the time of the trial], Meiji Pharma Spain, S.A., Madrid, Spain), whose biocompatibility and anti-inflammatory effects in the osteoarthritic joint have been previously reported [23]. Patients were followed for 12 months, with a safety check by phone at 3 months and in-person follow-up visits at 6 and 12 months. Those who met predefined retreatment criteria at the 6-month visit were offered a second injection. Retreatment criteria required: (1) a reduction in pain from baseline on the visual analog scale (VAS) below the minimal clinically important improvement (MCII) threshold; (2) the patient’s overall opinion; and (3) the absence of treatment-related adverse events after the first injection. Overall, 14 of 98 patients in the mITT population (14.3%) met these criteria and received a second 2% SH injection at 6 months; in this subgroup, the mean pain reduction from baseline to 12 months was 24.0%, with 50% achieving the MCII.

Patients eligible for the study were men or women aged 45 years or older with primary knee OA, confirmed by the American College of Rheumatology criteria, and radiographic evidence of grade II or III OA, as defined by the Kellgren–Lawrence scale. Patients had to report knee pain lasting at least 3 months before enrollment and score between 40 mm and 80 mm on a 100 mm pain VAS for the target knee. Only patients with a BMI < 30 kg/m^2^ were eligible for inclusion. Key exclusion criteria included marked knee inflammation, previous surgery on the affected knee, secondary causes of OA, and significant comorbidities such as metabolic or inflammatory joint diseases.

The mITT population included 98 patients who received 2% SH and completed at least one post-baseline assessment. The PP population comprised 74 patients who completed the 12-month follow-up without major protocol deviations.

### 2.2. Efficacy Outcomes

In the present study, efficacy outcomes were compared at baseline, 6 months, and 12 months in the mITT and PP populations.

The primary efficacy outcome was the reduction in knee pain intensity, measured by a VAS. In the SOYA Trial [20], pain was recorded on a 0–100 mm scale, where 0 indicates no pain and 100 represents the worst imaginable pain. Although the Trueba Davalillo study [19] originally collected pain intensity data on a 0–10 cm scale, all pain scores were standardized to a 0–100 mm scale for the non-inferiority exploratory analysis, consistent with the established correspondence between VAS and Likert-based measures of pain and function in OA [24] (transformed score = [100 × (observed value − minimum possible value)/(maximum possible value − minimum possible value)], where Minimum possible value = 0; Maximum possible value = 10; and Range = 10 − 0 = 10). 

Secondary efficacy outcomes of this study included changes in Western Ontario and McMaster Universities Osteoarthritis Index (WOMAC) scores and the proportion of patients achieving the MCII.

In the SOYA Trial [20], all WOMAC items were scored on a 0–100 VAS. The total possible score ranged from 0 to 500 for pain (5 items), 0 to 200 for stiffness (2 items), and 0 to 1700 for physical function (17 items), with higher scores indicating worse symptoms. In the Trueba Davalillo study [19], WOMAC items were rated on a five-point Likert scale (0–4), with total scores ranging from 0 to 96. The pain subscale ranged from 0 (less pain) to 20 (more pain), the stiffness subscale from 0 (less stiffness) to 8 (more stiffness), and the physical function subscale from 0 (better function) to 68 (worse function). As for the primary outcome, all WOMAC scores from the Trueba Davalillo study [19] were standardized to a 0–100 scale for the secondary analyses in the present study (transformed score = [100 × (observed value − minimum possible value for each domain)/(maximum possible value for each domain − minimum possible value for each domain)]).

The MCII, defined as the proportion of patients achieving an absolute improvement of ≥15 mm from baseline [25], was assessed for both the pain VAS and the WOMAC physical function subscale.

### 2.3. Non-Inferiority Margin

The exploratory, non-inferiority hypothesis in this indirect cross-trial analysis tested whether the efficacy of the 2% SH treatment (assessed by the relative change in pain intensity on the VAS after 12 months) was non-inferior to that of the 1% SH treatment, within a predefined margin. The non-inferiority margin defines the largest clinically acceptable loss of efficacy that still allows the experimental treatment to be considered non-inferior to the control [26]. In this study, a non-inferiority margin of −8% was established. This means that the lower bound of the 95% confidence interval for the difference in efficacy between treatments (2% minus 1% SH) should remain above −8% for the 2% SH to be considered non-inferior.

The choice of an exploratory −8% margin was based on data supporting approximately 8% (corresponding to 8 mm on a 0–100 mm scale) as a clinically acceptable difference in pain improvement for non-inferiority in OA trials [27,28,29]. The non-inferiority framework was applied for exploratory purposes, recognizing that the indirect comparison from a cross-trial analysis and the consequent absence of randomization between treatment groups preclude definitive causal inference and cannot rule out residual confounding.

### 2.4. Post Hoc Assessment of Sample Size Adequacy

Because this analysis combines data from two previously completed trials with fixed sample sizes (*n* = 97 and *n* = 98), no independent, prospective sample-size calculation was performed for the present cross-trial comparison. Instead, we conducted a retrospective assessment of the precision and power afforded by the already-available sample.

The primary endpoint of this study was the percentage change in VAS-assessed knee pain from baseline to 12 months in patients treated with 1% or 2% SH. In the SOYA Trial [20], the mean percentage reduction in pain intensity at 12 months with 2% SH was 37.7% (standard deviation = 50.6). In the Trueba Davalillo study [19], patients treated with 1% SH showed a mean reduction of 33.6% (standard deviation = 12.4). Using these observed standard deviations, an assumed true difference of 4% in favor of the 2% SH group, and the predefined non-inferiority margin of −8%, a sample size of approximately 97–98 patients per group corresponded to an estimated post hoc power of approximately 62% at a one-sided significance level of 2.5%, consistent with the exploratory nature of this indirect comparison.

### 2.5. Statistical Methods

Quantitative variables were summarized using descriptive statistics. To evaluate treatment effects over time, median scores for each outcome measure were summarized at baseline, 6 months, and 12 months, and compared between treatment groups at each time point. In addition, the relative change (%) in scores was calculated at 6 and 12 months using the formula: [(baseline score − follow-up score)/baseline score] × 100.

Between-group comparisons (2% vs. 1% SH) for pain intensity scores and WOMAC scores were performed using the Mann–Whitney U/Fisher’s exact test. In addition, an analysis of covariance (ANCOVA) was performed to compare relative changes between groups at 6 and 12 months, adjusting for potential baseline differences between the populations of the SOYA and the Trueba Davalillo studies. The ANCOVA model included the baseline value of each outcome as a covariate. Available baseline demographic and clinical variables that showed a *p*-value < 0.20 in univariate analyses were also included in the ANCOVA model. These included age, body mass index, sex, Kellgren–Lawrence grade, and paracetamol use during the study. An exploratory, post hoc comparative analysis between patients treated with 2% SH in the SOYA trial and those treated with betamethasone in the Trueba Davalillo study was also conducted using ANCOVA. This analysis assessed the differences in relative change in pain and in physical function outcomes at 12 months.

For both pain intensity and the WOMAC physical function subscale, dichotomous variables were created to classify patients as having achieved or not achieved the MCII at 6 and 12 months. Between-group comparisons (2% vs. 1% SH) were performed using the chi-square test or Fisher’s exact test, as appropriate.

For the primary non-inferiority endpoint, a one-sided significance level of 0.025 was applied. The 95% confidence interval for the corresponding between-group mean difference, used to evaluate the non-inferiority margin, was calculated using an unequal-variances (Welch–Satterthwaite) approach. A significance level of 0.05 was used for all other analyses. Missing post-baseline assessments for the primary and secondary efficacy outcomes in the mITT population were imputed using the last observation carried forward method; no imputation was performed for other variables.

## 3. Results

### 3.1. Baseline Demographics and Clinical Characteristics

Table 1 summarizes the baseline demographic and clinical characteristics of the mITT population.

Patients treated with 2% SH were significantly older (median age 69.0 years; 66.3% ≥ 65 years) than those treated with 1% SH (median age 62.0 years; 32% ≥ 65 years). Women predominated in both groups (73.5% in the 2% SH cohort and 60.8% in the 1% cohort).

The median BMI was slightly higher in the 1% SH group (28.8 kg/m^2^ vs. 27.4 kg/m^2^). Most patients in both groups had a BMI > 25 kg/m^2^, with a higher obesity (BMI ≥ 30) rate in the 1% group (40.2%) and a predominance of overweight (BMI ≥ 25) patients in the 2% group (77.6%). Underweight patients (1%) were only observed in the 2% group.

Regarding the painful side, 12.4% of patients treated with 1% SH reported bilateral knee involvement, whereas all patients treated with 2% SH had unilateral involvement. The involvement of the right and left sides was similar across studies (46.4% and 41.2%, respectively, in the 1% SH group and 57.1% and 42.9%, respectively, in the 2% SH study).

Kellgren–Lawrence grading showed a higher proportion of patients with moderate joint damage (grade III) in the 2% SH cohort (56.15% vs. 36.1% in the 1% SH cohort). Conversely, most patients in the 1% SH cohort were classified as grade II (63.9% vs. 43.9% in the 2% SH group). Finally, the use of paracetamol as concomitant medication was more frequent in patients treated with 1% SH (68% vs. 41.8%).

A similar pattern of baseline characteristics was observed in the PP population, as shown in Supplementary Table S2.

### 3.2. Non-Inferiority Analysis

**Figure 1:**
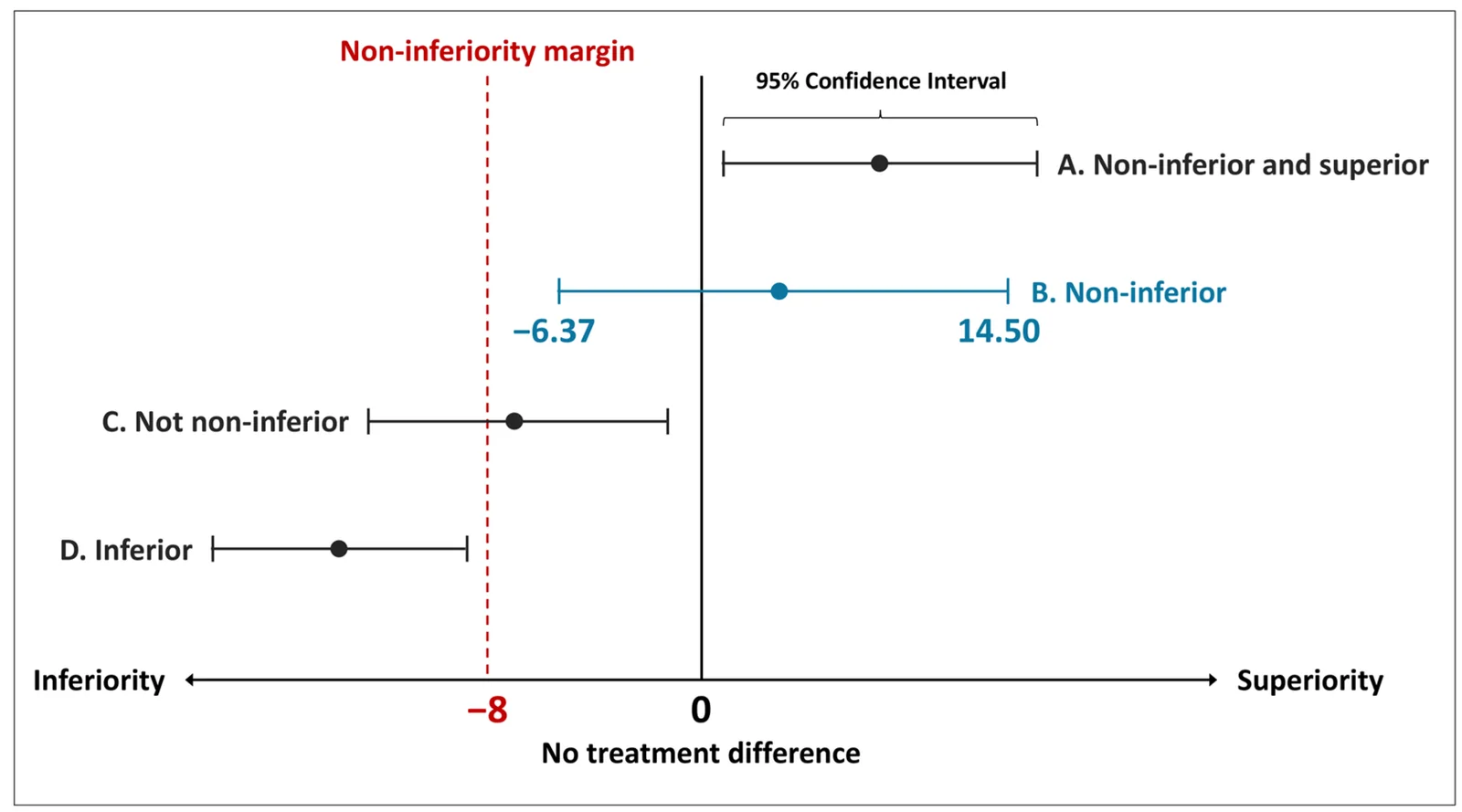
Different findings that can result from trials with a non-inferiority hypothesis test. (A) Hypothetical scenario in which the result confirms non-inferiority between treatments and allows for superiority testing, with the entire 95% confidence interval for the difference between treatments above the no-treatment-difference line. (B) Result obtained in the present study, with non-inferiority between treatments observed as the lower limit of the 95% confidence interval for the difference between treatments remaining above the predefined non-inferiority margin. (C) Hypothetical scenario with an inconclusive result for non-inferiority, as part of the 95% confidence interval of the difference between treatments falls within the established non-inferiority margin. (D) Hypothetical scenario in which the entire 95% confidence interval of the difference between treatments surpasses the non-inferiority margin, confirming the inferiority of one of the treatments.

At 12 months, the mean relative change from baseline in pain intensity was 33.61% in the mITT population treated with 1% SH and 37.67% in the group treated with 2% SH (Table 2, Figure 1). The mean difference between groups was 4.06%. The 95% confidence interval ranged from −6.37% to 14.50%, with the lower bound of the confidence interval (−6.37%) remaining above the −8% margin. A similar result was observed in the PP population (Supplementary Table S3). The mean difference between groups in the PP population was 7.28% (95% confidence interval: −3.71% to 18.27%), with the lower bound of the confidence interval remaining within the predefined non-inferiority margin. These findings are compatible with non-inferiority between treatments in this exploratory cross-trial analysis.

### 3.3. Efficacy Analysis

#### 3.3.1. Pain Intensity Scores

**Figure 2:**
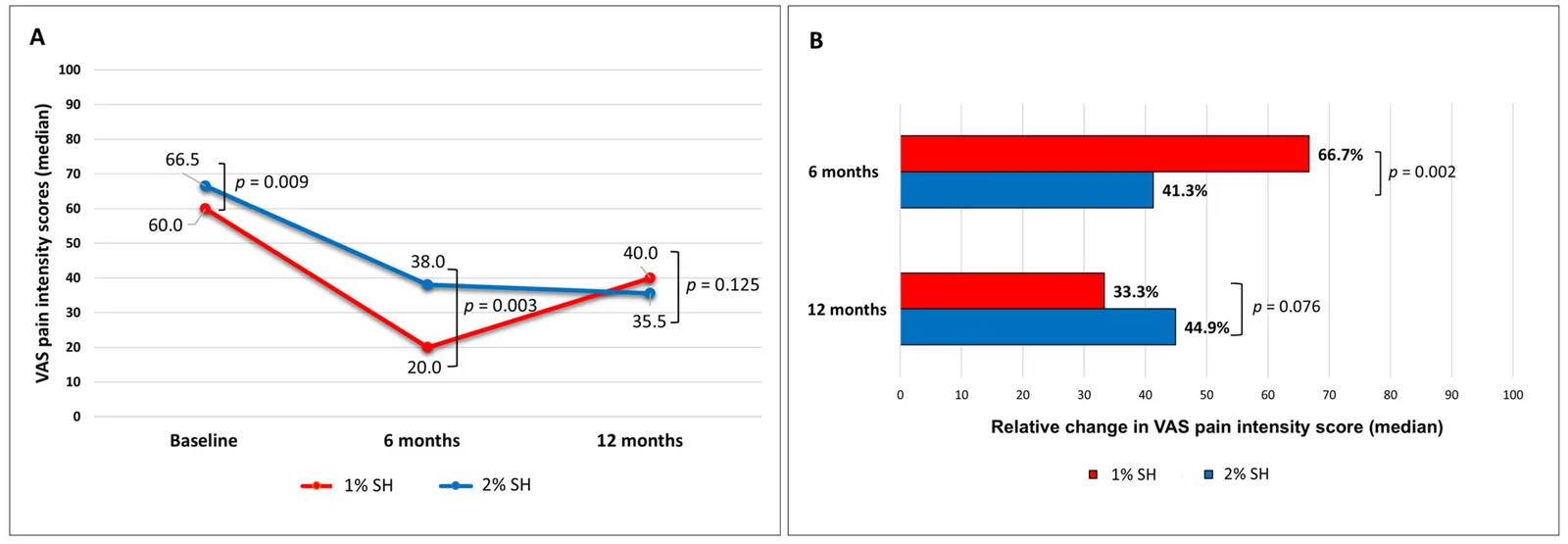
Median pain intensity scores (measured by the VAS) in the mITT population. (**A**) Median pain intensity scores from baseline to 12 months of follow-up. (**B**) Median relative change (%) in pain intensity scores from baseline at 6 and 12 months. *p*-values were calculated using the Mann–Whitney U test. SH: sodium hyaluronate; VAS: visual analog scale.

Figure 2A shows the median pain intensity scores in the mITT population from baseline to 12 months of follow-up. Although statistically significant differences were observed between the 1% and 2% SH groups at baseline (60.0 mm [Q1–Q3: 60.0–60.0] vs. 66.5 mm; [Q1–Q3: 53.8–73.0]; *p* = 0.009) and at 6 months (20.0 mm [Q1–Q3: 20.0–30.0] vs. 38.0 mm; [Q1–Q3: 11.5–60.5] *p* = 0.003), no significant difference was found at 12 months (40.0 mm [Q1–Q3: 40.0–40.0] vs. 35.0 mm [Q1–Q3: 10.0–70.0]; *p* = 0.125). Figure 2B presents the relative change in pain intensity scores at 6 and 12 months compared to baseline. At 6 months, the median relative reduction in pain was significantly greater in patients treated with 1% SH (66.7% [Q1–Q3: 50.0–66.7] vs. 41.3% [Q1–Q3: 0–80.1]; *p* = 0.002). However, by 12 months, no statistically significant difference was observed between the two cohorts (33.3% [Q1–Q3: 28.6–42.9] vs. 44.9% [Q1–Q3: −6.8–85.4]; *p* = 0.076). A similar pattern was observed in the PP population (Supplementary Figure S1).

As shown in Table 3 (mITT population) and Supplementary Table S4 (PP population), multivariate analysis (ANCOVA) showed no significant difference in relative pain reduction at 12 months between groups after adjusting for baseline pain scores. Similarly, after adjusting for baseline demographic and clinical variables with a significance level of *p* < 0.20 in previous analyses, no significant difference was found between 1% and 2% SH groups. These findings show that when the relevant baseline differences between the populations of both studies are taken into account in statistical analysis, no difference in efficacy arises between treatments for all evaluated outcomes in the mITT population.

#### 3.3.2. WOMAC Scores

**Figure 3:**
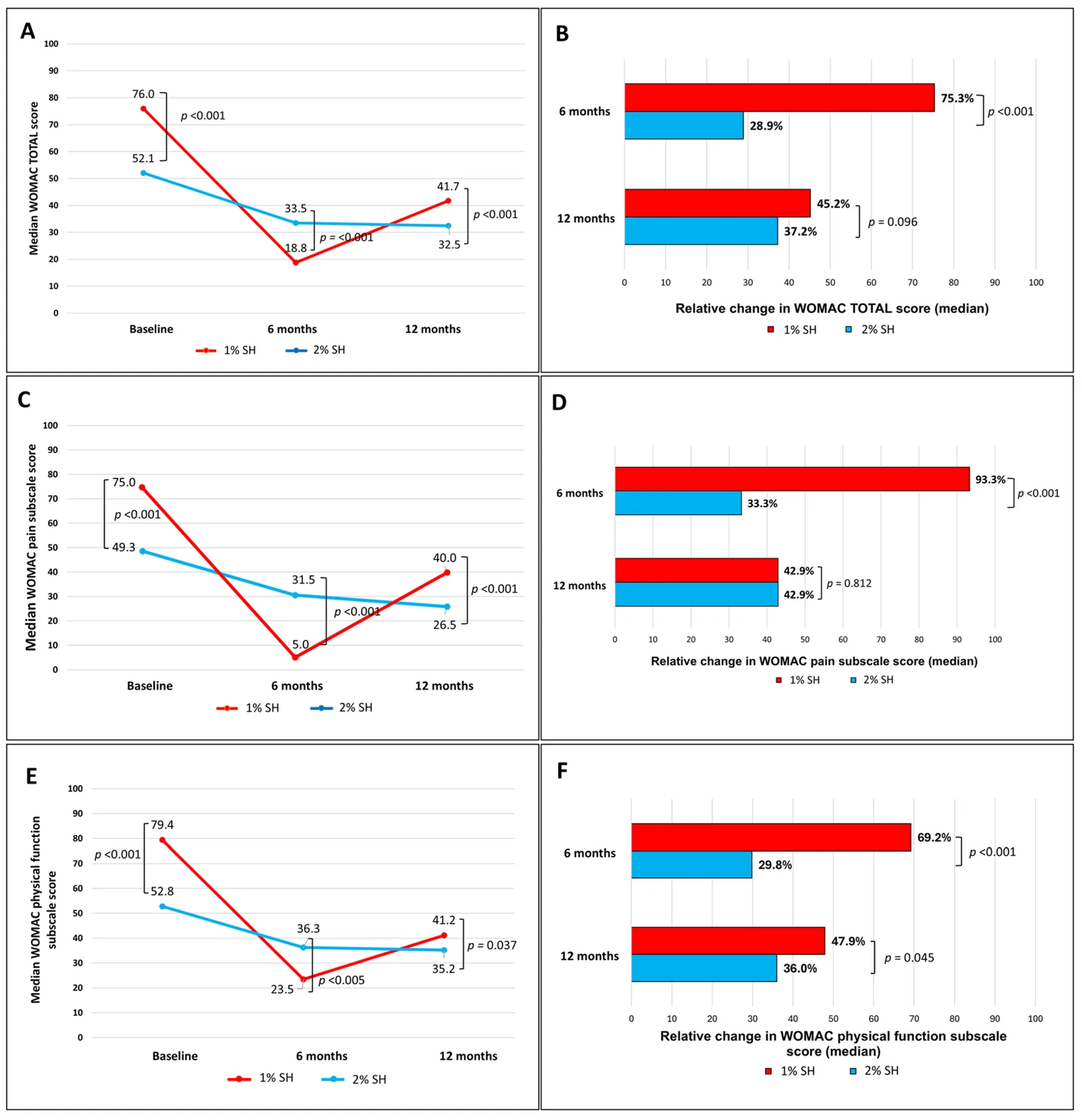
Median WOMAC scores (**A**,**C**,**E**) and median relative change from baseline (**B**,**D**,**F**) for total score (**A**,**B**), pain subscale (**C**,**D**), and physical function subscale (**E**,**F**) in the mITT population. *p*-values were calculated using the Mann–Whitney U test. WOMAC: Western Ontario and McMaster Universities Osteoarthritis Index; SH: sodium hyaluronate.

As shown in Figure 3A, the median total WOMAC score decreased in both groups over the 12-month period. At baseline, the mITT population treated with 1% SH had significantly higher median scores than those in the 2% SH group (76.0 points [Q1–Q3: 74.0–80.2] vs. 52.1 points [Q1–Q3: 39.5–64.6]; *p* < 0.001). However, at 6 months, this trend reversed; the cohort treated with 1% SH showed a lower median score (18.8 [Q1–Q3: 17.7–20.8]) than the group treated with 2% SH (33.5 [Q1–Q3: 12.2–49.2]; *p* < 0.001). By 12 months, the pattern shifted again, with the 1% SH group presenting higher median scores (41.7 [Q1–Q3: 39.1–43.8] vs. 32.5 [Q1–Q3: 9.4–51.0]; *p* < 0.001). When analyzing the median relative change in the total WOMAC score (Figure 3B), a significantly greater reduction was observed in the mITT population treated with 1% SH at 6 months (75.3% [Q1–Q3: 73.3–77.1] vs. 28.9% [Q1–Q3: 0–68.4]; *p* < 0.001). However, by 12 months, the difference was no longer statistically significant (45.2% [Q1–Q3: 41.1–50.0] vs. 37.2% [Q1–Q3: 4.9–75.5]; *p* = 0.096).

A similar pattern was observed for the WOMAC pain subscale. As illustrated in Figure 3C, median baseline scores were higher in the mITT population treated with 1% SH (75.0 [Q1–Q3: 65.0–85.0] vs. 49.3 [Q1–Q3: 37.3–62.3]; *p* < 0.001). At 6 months, patients treated with 1% SH had significantly lower median pain scores (5.0 [Q1–Q3: 5.0–5.0]) than patients treated with 2% SH (31.5 [Q1–Q3: 10.1–46.7]; *p* < 0.001). At 12 months, patients treated with 1% SH presented higher median pain scores than patients treated with 2% SH (40.0 [Q1–Q3: 35.0–50.0] vs. 26.5 [Q1–Q3: 9.0–46.3]; *p* < 0.001). The median relative reduction in the WOMAC pain subscale (Figure 3D) was greater in patients treated with 1% SH at 6 months (93.3% [Q1–Q3: 90.5–94.7] vs. 33.3% [Q1–Q3: 0–71.7]; *p* < 0.001), with no significant difference at 12 months (42.9% [Q1–Q3: 33.3–60.0] vs. 42.9% [Q1–Q3: −4.45–77.6]; *p* = 0.812).

As shown in Figure 3E, baseline median scores for the WOMAC physical function subscale were also higher in the mITT population treated with 1% SH (79.4 [Q1–Q3: 73.5–82.4] vs. 52.8 [Q1–Q3: 40.1–65.3]; *p* < 0.001). At 6 months, this trend reversed, with significantly lower median scores in patients treated with 1% SH (23.5 [Q1–Q3: 22.1–26.5] vs. 36.3 [Q1–Q3: 11.2–55.6]; *p* < 0.005). By 12 months, median scores were 41.2 points [Q1–Q3: 36.8–44.1] in patients treated with 1% SH and 35.2 points [Q1–Q3: 8.2–53.1] in those treated with 2% SH (*p* = 0.037). At 6 months, the median relative improvement in WOMAC physical function was substantially greater in patients treated with 1% SH (69.2% [Q1–Q3: 66.0–72.6] vs. 29.8% [Q1–Q3: 0–70.1]; *p* < 0.001). At 12 months, the difference between groups persisted, with median improvements of 47.9% [Q1–Q3: 42.7–52.5] in the 1% cohort and 36% [Q1–Q3: 4.0–75.8] in the 2% cohort (*p* = 0.045) (Figure 3F). A similar pattern was observed for WOMAC results in the PP population (Supplementary Figure S2).

Multivariate analysis showed no statistically significant differences between the 1% and 2% SH groups in the relative change of total WOMAC scores or WOMAC physical function subscale scores at 12 months, either in the mITT (Table 3) or PP populations (Supplementary Table S4). A statistically significant difference in the WOMAC pain subscale scores was observed in the PP population (*p* = 0.044), but this difference was not confirmed in the mITT population.

#### 3.3.3. Minimal Clinically Important Improvement (MCII)

**Figure 4:**
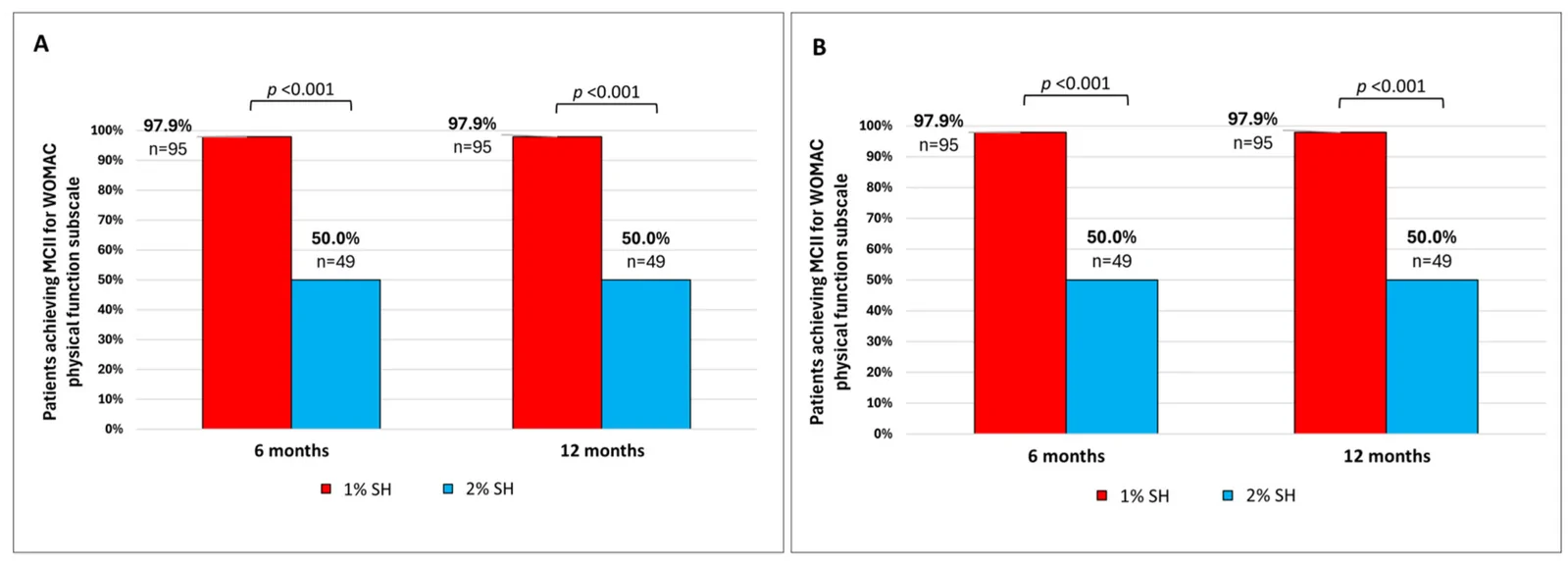
Proportion of the mITT population achieving the minimal clinically important improvement (MCII) for pain intensity (**A**) and WOMAC physical function (**B**) at 6 and 12 months. The MCII was defined as the proportion of patients achieving an absolute improvement of ≥15 mm from baseline. *p*-values were calculated using Fisher’s exact test. SH: sodium hyaluronate.

For pain intensity, a significantly greater proportion of patients in the 1% SH group achieved the MCII threshold at 6 months in the mITT population (95/97, 97.9% vs. 59/98, 60.2%; risk difference −37.7%, 95% CI: −47.7% to −27.3%); risk ratio 0.615, 95% CI: 0.52 to 0.72); *p* < 0.001; Figure 4A). At 12 months, this difference remained significant in the mITT analysis (75/97, 77.3% vs. 61/98, 62.2%; risk difference −15.1%, 95% CI: −27.3% to −2.2%; risk ratio 0.805, 95% CI: 0.67 to 0.97; *p* < 0.05; Figure 4A). For WOMAC physical function, 95/97 (97.9%) of the mITT population treated with 1% SH achieved MCII at 6 months, compared to 49/98 (50.0%) in the 2% SH group (risk difference −47.9%, 95% CI: −57.8% to −36.9%; risk ratio 0.511, 95% CI: 0.42 to 0.62); *p* < 0.001; Figure 4B). At 12 months, the proportion remained significantly higher in the 1% SH group (95/97, 97.9% vs. 49/98, 50.0%; risk difference −47.9%, 95% CI: −57.8% to −36.9%; risk ratio 0.511, 95% CI: 0.42 to 0.62; *p* < 0.001; Figure 4B).

A similar pattern of between-group differences was observed in the PP population (Supplementary Figure S3).

#### 3.3.4. Post Hoc Comparison of 2% SH Versus Betamethasone

In this exploratory, post hoc analysis, the mITT population treated with 2% SH showed a significantly greater relative improvement in pain intensity and WOMAC physical function at 12 months compared to those treated with betamethasone (Table 4).

For pain intensity scores, the adjusted mean relative change was 37.7% in the 2% SH group vs. 8.2% in the betamethasone group (*p* < 0.001). For WOMAC physical function scores, the adjusted mean relative change was 30.3% in the 2% SH group vs. 13.2% in the betamethasone group (*p* < 0.001). Because the relative-change formula divides by the baseline score, patients with a low baseline WOMAC physical function score can show disproportionately large percentage changes when their score worsens during follow-up, even when the absolute change is modest; this accounts for the wide range of relative-change values observed in the 2% SH group (Table 4), including the minimum value of −199.7%. Similar results were obtained in the PP population (Supplementary Table S5).

## 4. Discussion

This study investigated whether intra-articular 2% SH is non-inferior to 1% SH for managing knee OA. In this condition, where no disease-modifying therapies exist and available treatments focus primarily on symptom relief [30], non-inferiority analyses are essential to establish whether a new treatment option, with potential advantages beyond efficacy, achieves sufficient effectiveness to be clinically useful [31]. Such evidence enables clinicians to consider additional factors such as tolerability, adherence, and healthcare resource utilization when making treatment decisions [32]. In this context, indirect cross-trial comparisons can provide useful evidence when direct head-to-head studies are unavailable [33].

A key element of non-inferiority studies is the definition of a clinical margin, which specifies the maximum acceptable loss of efficacy compared to the standard treatment. This study set the non-inferiority margin at −8%, consistent with thresholds validated in OA trials comparing SH with other formulations. In these trials, an 8 mm difference on a 100 mm VAS for WOMAC pain was considered clinically meaningful [27,28,29].

Both studies included in this analysis [19,20] enrolled patients with radiographic grade II–III knee OA and used standardized outcome measures, enabling meaningful comparisons. As expected in an indirect comparison, baseline differences between the two populations were observed, particularly in age, BMI, Kellgren–Lawrence grade, bilateral knee involvement, and paracetamol use. This comparison is further limited by asymmetric trial design—the Trueba Davalillo study [19] was randomized and controlled, whereas the SOYA Trial [20] was uncontrolled, so no common comparator links the two cohorts, and this should be regarded as a naïve and exploratory cross-trial comparison rather than a randomized head-to-head design. Even though multivariable ANCOVA models were applied to adjust for these differences and mitigate potential confounding, it is important to acknowledge that such adjustments cannot replicate the balance achieved through randomization, nor can they account for unmeasured confounders such as differences in clinical practice, healthcare setting, patient expectations between countries, disease duration, or prior treatment history beyond the specified exclusion criteria. Therefore, residual confounding cannot be excluded and should be considered when interpreting comparative outcomes.

The primary result of our study supports the non-inferiority of 2% SH relative to 1% SH at 12 months of follow-up, based on the relative reduction in pain intensity. Secondary analyses were broadly consistent with the primary findings. Although transient differences favoring 1% SH were observed at 6 months for pain intensity and WOMAC outcomes, these differences were no longer significant after adjustment at 12 months, suggesting similar improvements in pain, physical function, and overall symptoms over one year.

A higher proportion of patients in the 1% SH group achieved the MCII threshold for both pain and WOMAC physical function at 6 and 12 months. However, this finding was not mirrored in the adjusted continuous analyses, which showed no significant between-group differences at 12 months. The MCII findings should be interpreted in light of important methodological considerations. The MCII—and its underlying construct, the Minimal Clinically Important Difference (MCID)—is derived from dichotomization of continuous outcomes and is highly sensitive to baseline severity, direction of change, and calculation methods [34,35,36]. Such methodological variability limits the reliability of MCII for cross-trial comparisons. Additionally, the two cohorts differed substantially at baseline in the specific outcomes used to define MCII: 1% SH patients started with worse WOMAC physical function scores (median 79.4 vs. 52.8) but better (lower) pain scores (median 60.0 mm vs. 66.5 mm) than 2% SH patients. This asymmetry means that a simple ‘more room to improve’ explanation is plausible for the WOMAC function MCII discordance but does not account for the pain MCII discordance, where the group with less room to improve (1% SH) nonetheless showed substantially higher responder rates. Confirming this would require an adjusted responder analysis, which was not part of the original statistical analysis plan; we acknowledge this as a limitation of the present analysis. Finally, the MCII threshold applied was derived from a large multinational cohort [25] but was not separately validated for Likert-derived versus native VAS-based WOMAC formats. The MCII findings should therefore be interpreted with appropriate caution.

Comparison with betamethasone provides additional exploratory context on the value of hyaluronic acid therapy. In the randomized trial by Trueba Davalillo et al. [19], patients treated with 1% SH or betamethasone improved from baseline to 12 months, but the magnitude of benefit was substantially greater with SH. Global pain decreased by 33% with 1% SH vs. 9% with betamethasone, and WOMAC physical function improved by 47% vs. 13%, respectively. In our post hoc, hypothesis-generating analysis, a greater adjusted mean improvement was also observed with 2% SH relative to betamethasone (pain intensity: 37.7%, 95% CI 27.5–47.8, vs. 8.2%, 95% CI 5.2–11.1; WOMAC physical function: 30.3%, 95% CI 19.3–41.4, vs. 13.2%, 95% CI 1.4–14.9). Taken together, these findings suggest that SH formulations may provide greater symptom relief than intra-articular corticosteroids after 12 months of treatment.

Beyond efficacy, the choice between 1% and 2% SH may be influenced by practical and contextual considerations. The 2% formulation, given as a single injection with the option of a booster at 6 months, may offer practical advantages by reducing treatment burden, which could facilitate adherence [37,38], although this was not directly assessed in either trial or in the present study. Conversely, the 1% formulation, administered as a series of weekly injections, may be preferred in scenarios where closer clinical follow-up is desirable or early symptom relief is prioritized. Our results indicate that both formulations are effective long-term options for managing knee OA. Thus, treatment choice may ultimately be guided by patient preferences, logistical considerations, and physician judgment. Regardless of the SH concentration selected, treatment is typically most effective when combined with structured conservative management, such as therapeutic exercise programs, which have been shown to improve pain and function in primary knee OA [39].

Regarding safety, both original studies reported favorable tolerability of SH. However, adverse events were reported according to each study protocol, and no formal harmonization of adverse event definitions or coding was performed to allow a direct comparative analysis. Therefore, safety findings should be interpreted descriptively rather than as evidence of comparative safety between formulations. The most frequently reported adverse events were transient local reactions, including injection-site pain, joint swelling, mild synovial effusion, and temporary stiffness, all of which resolved spontaneously within a few days [19,20]. Importantly, no unexpected or serious treatment-related adverse events were reported in either study. These findings align with the well-tolerated safety profile of intra-articular hyaluronic acid [40]. This is particularly relevant in older adults with comorbidities, who represent most of the patients with knee OA [41,42].

Several methodological aspects strengthen this analysis. Both studies enrolled patients with symptomatic, radiographically confirmed grade II–III knee OA. Robust statistical analyses were conducted in both mITT and PP populations, yielding consistent results. Nonetheless, this study does not constitute a prospectively designed randomized non-inferiority trial. The comparison is indirect and based on independent studies conducted in different geographic settings (Mexico and Spain), with slightly different inclusion criteria, including variations in body mass index thresholds (≤35 kg/m^2^ versus ≤30 kg/m^2^) and minimum age requirements (≥40 versus ≥45 years). These differences may limit generalizability to broader populations with knee OA, particularly to patients with more severe obesity or distinct demographic profiles.

Baseline pain severity also differed significantly between cohorts, with lower median pain scores in the 1% SH group than in the 2% SH group (60.0 vs. 66.5 mm). Because baseline symptom severity may influence the magnitude of observed improvement, this imbalance represents an additional potential source of confounding. Baseline pain was included as a covariate in the ANCOVA model to mitigate this potential bias; however, residual confounding cannot be excluded.

An additional limitation relates to the open-label design of both original studies, as the absence of blinding may introduce performance and reporting bias, particularly when outcomes are patient-reported and subjective, such as VAS pain scores and WOMAC measures. Although patient-reported outcome measures are well-established and validated tools for assessing pain and function in knee OA [24,43,44], patient expectations regarding treatment regimen (multiple versus single injection) may also influence perceived symptom improvement. While this limitation applies to both source trials, it may still contribute to variability in treatment response and should be taken into account when evaluating comparative findings. Moreover, because retreatment with a second 2% SH injection at 6 months was offered specifically to patients who had not yet achieved the MCII [20], the 12-month outcome in the 2% SH group reflects a mixture of single- and two-injection recipients. Although only 14.3% of patients received a second injection, this additional intervention may have influenced subsequent outcomes through a treatment-specific effect or a renewed contextual/placebo response. This may have contributed, at least partially, to the temporal pattern observed in several analyses, in which outcomes favored 1% SH at 6 months but became more comparable between groups at 12 months. Because retreatment was triggered by insufficient interim response rather than randomly assigned, its specific contribution cannot be disentangled from the underlying clinical trajectory. Accordingly, the 12-month comparison should not be interpreted as reflecting a strictly single-injection regimen.

Another important factor to consider is the transformation of the pain and WOMAC scores originally collected using Likert-type scales in the Trueba Davalillo study [19] into VAS equivalents. While this approach could be viewed as a limitation, prior research in OA has demonstrated a strong correlation and comparable discriminatory precision between Likert and VAS measures [24], supporting its use in cross-trial analyses. We acknowledge, however, that numerical harmonization of scales does not fully eliminate potential measurement heterogeneity between the two instruments. Finally, the fixed sample size yielded an estimated post hoc statistical power of approximately 62%. Although the primary 95% confidence interval remained above the predefined non-inferiority margin, this limited power reduces precision and warrants caution when interpreting non-significant between-group differences and the overall robustness of the findings. When taken together, these limitations indicate that this study should be interpreted as hypothesis-generating rather than confirmatory evidence of comparative efficacy.

## 5. Conclusions

This exploratory cross-trial analysis provides hypothesis-generating evidence compatible with non-inferiority of 2% SH relative to 1% SH in improving pain and physical function over 12 months in patients with knee OA. These findings require confirmation in adequately powered randomized head-to-head non-inferiority trials.

## Figures and Tables

**Table 1:** Baseline demographics and clinical characteristics of the mITT population.

Characteristics		1% SH Trueba Davalillo Study (*N* = 97)	2% SH SOYA Trial (*N* = 98)	*p*-Value
Age, median ± (Q1–Q3) (years)		62.0 (59.0–65.5)	69.0 (62.5–74.3)	<0.001 ^1^
Age, n (%)	<65 years	66 (68.0)	33 (33.7)	<0.001 ^2^
	≥65 years	31 (32.0)	65 (66.3)	
Sex, n (%)	Male	38 (39.2)	26 (26.5)	0.068 ^2^
	Female	59 (60.8)	72 (73.5)	
BMI, median ± (Q1–Q3) (kg/m^2^)		28.8 (25.0–32.0)	27.4 (25.2–29.4)	0.008 ^1^
BMI category, n (%)	Underweight: BMI < 18.5 kg/m^2^	0 (0.0)	1 (1.0)	<0.001 ^2^
	Normal: BMI 18.5–24.99 kg/m^2^	23 (23.7)	19 (19.4)	
	Overweight: BMI 25–29.99 kg/m^2^	35 (36.1)	76 (77.6)	
	Obese: BMI ≥ 30 kg/m^2^	39 (40.2)	2 (2.0)	
Painful side, n (%)	Bilateral	12 (12.4)	0 (0.0)	<0.001 ^2^
	Right	45 (46.4)	56 (57.1)	
	Left	40 (41.2)	42 (42.9)	
K&L Scale, n (%)	II	62 (63.9)	43 (43.9)	0.006 ^2^
	III	35 (36.1)	55 (56.1)	
Paracetamol consumption, n (%)	No	31 (32.0)	57 (58.2)	<0.001 ^2^
	Yes	66 (68.0)	41 (41.8)	

BMI: body mass index; K&L: Kellgren–Lawrence; SH: sodium hyaluronate. ^1^: *p*-value analyzed using the Mann–Whitney test. ^2^: *p*-value analyzed using Fisher’s exact test.

**Table 2:** Relative change in pain intensity from baseline to 12 months in the mITT population.

Measure (%)	1% SH Trueba Davalillo Study (*N* = 97)	2% SH SOYA Trial (*N* = 98)
Mean	33.61	37.67
Standard deviation	12.43	50.63
Standard error	1.26	5.11
Mean difference	4.06	
Standard error of the difference	5.29	
95% confidence interval	−6.37; 14.50	

SH: sodium hyaluronate.

**Table 3:** Multivariate analysis (ANCOVA) of the relative change in pain intensity (measured by VAS) and WOMAC scores at 12 months in the mITT population.

Outcome	Adjusted Mean Relative Change (95% CI) 1% SH Trueba Davalillo Study (*N* = 97)	Adjusted Mean Relative Change (95% CI) 2% SH SOYA Trial (*N* = 98)	*p*-Value
Pain intensity (measured by VAS) adjusted for baseline pain scores	34.56 (27.29–41.84)	36.73 (29.49–43.96)	0.679
Pain intensity (measured by VAS) adjusted for baseline variables	33.45 (25.94–40.97)	37.83 (30.36–45.30)	0.445
Total WOMAC scores adjusted for baseline scores	40.40 (30.97–49.83)	37.61 (28.25–46.98)	0.723
Total WOMAC scores adjusted for baseline variables	39.76 (30.24–49.28)	38.24 (28.80–47.69)	0.852
WOMAC pain subscale scores adjusted for baseline scores	31.73 (22.31–41.16)	43.02 (33.66–52.38)	0.140
WOMAC pain subscale adjusted for baseline variables	32.05 (22.51–41.59)	42.70 (33.23–52.18)	0.180
WOMAC physical function subscale scores adjusted for baseline scores	42.47 (32.86–52.07)	35.30 (25.76–44.84)	0.366
WOMAC physical function subscale adjusted for baseline variables	41.73 (31.95–51.52)	36.02 (26.31–45.70)	0.492

VAS: visual analog scale; WOMAC: Western Ontario and McMaster Universities Osteoarthritis Index; CI: confidence interval; SH: sodium hyaluronate.

**Table 4:** Post hoc, exploratory comparison of relative change in VAS pain and WOMAC physical function scores at 12 months in the mITT population treated with 2% SH (SOYA trial) or betamethasone (Trueba Davalillo study).

Outcome	Group	Adjusted Mean Relative Change (95% CI)	SD	Min	Max	*N*	*p*-Value
Pain intensity (measured by VAS)	Betamethasone (Trueba Davalillo study)	8.2 (5.2–11.1)	14.8	−50.0	62.5	98	<0.001
	2% SH (SOYA trial)	37.7 (27.5–47.8)	50.6	−95.7	100.0	98	
WOMAC physical function	Betamethasone (Trueba Davalillo study)	13.2 (1.4–14.9)	8.8	−2.7	48.9	98	<0.001
	2% SH (SOYA trial)	30.3 (19.3–41.4)	55.1	−199.7	99.7	98	

## Data Availability

The datasets analyzed in this study derive from two independently sponsored clinical trials, each governed by its own data ownership agreements and informed consent provisions that did not anticipate third-party data sharing at the time of conduct. As such, the harmonized patient-level dataset is not publicly available, in accordance with sponsor policy and the original trials’ data governance terms. Reasonable requests for access to summary-level statistical outputs may be directed to the corresponding author and will be evaluated on a case-by-case basis, subject to sponsor approval and any applicable data-use agreements.

## Supplementary Materials

The following supporting information can be downloaded at: https://www.mdpi.com/article/10.3390/jcm15176571/s1.

## Abbreviations

The following abbreviations are used in this manuscript:

ANCOVA	Analysis of covariance
BMI	Body mass index
MCID	Minimal Clinically Important Difference
MCII	Minimal Clinically Important Improvement
mITT	Modified intention-to-treat
OA	Osteoarthritis
PP	Per protocol
SH	Sodium hyaluronate
VAS	Visual analog scale
WOMAC	Western Ontario and McMaster Universities Osteoarthritis Index
