# Supplementary material for: Exploratory Cross-Trial Non-Inferiority Analysis of 2% Versus 1% Sodium Hyaluronate in Knee Osteoarthritis
Source: J Clin Med. 2026 Aug 26;15(17):6571. doi: 10.3390/jcm15176571 (PMC13566129; doi:10.3390/jcm15176571)
Supplement: Supplementary file 1 [file jcm-15-06571-s001.zip › jcm-4432232-supplementary.pdf]

Table S1 - Methodological comparison of the two source trials

Characteristic	1% SH<br>Trueba Davalillo study	2% SH<br>SOYA Trial
**Design**	Prospective, randomized, open-label, parallel-group trial (SH vs. betamethasone); only the SH arm is used in the present comparison	Prospective, single-arm, multicenter, open-label study (no comparator arm)
**Country / setting**	Mexico; single center	Spain; 7 hospitals
**Recruitment period**	April 2008 – February 2011	January 2019 – May 2020
**Analysis populations (*n*)**	mITT SH = 97; PP SH = 89	mITT = 98; PP = 74 (12 months)
**Age**	40–85 years	≥45 years
**Radiographic severity (KL grade)**	Grade II–III	Grade II–III
**BMI restriction**	Excluded if BMI ≥35 kg/m² (obesity type I still eligible)	Excluded if BMI ≥30 kg/m² (any obesity excluded)
**Baseline pain criterion**	No specific VAS pain range required for inclusion	VAS pain 40–<80 mm in target knee; ≤20 mm in contralateral knee
**Formulation / molecular weight**	1% sodium hyaluronate, ~900,000 Da	2% sodium hyaluronate, ~1,000,000 Da
**Injection regimen**	5 injections: day 0 and weekly thereafter (2.5 mL each)	Single injection (98 mg), with optional retreatment at 6 months
**Retreatment**	Not applicable (fixed 5-injection regimen)	Optional 2nd injection at 6 months if criteria met (VAS improvement < MCII, patient opinion, no treatment-related AEs); 14/98 (14.3%) retreated
**Comparator arm**	Betamethasone	None (single-arm design)
**Follow-up schedule**	3, 6, 9, and 12 months (in-person visits)	Telephone contact at 3 months (safety only); in-person visits at 6 and 12 months
**Primary outcome**	Between-group difference in change from baseline in global pain (VAS) and WOMAC function at 12 months	Within-group change from baseline in VAS pain at 12 months (no comparator)
**Pain scale**	0–100 mm VAS	0–100 mm VAS
**WOMAC format**	5-point Likert scale (0–4 per item); subscales 0–20 (pain), 0–68 (function), 0–8 (stiffness)	VAS-based, normalized to 0–100
**Concomitant / co-administered medication**	Glucosamine sulfate + meloxicam (1 month), then glucosamine + chondroitin sulfate (1 additional month), given to all patients regardless of group; paracetamol permitted as rescue (≤3 g/day)	No standardized co-medication; paracetamol permitted as rescue (≤3 g/day), discontinued ≥24 h before visits
**Blinding**	Assessing physician blinded to group assignment; patients and injecting personnel not blinded	Open-label; no blinding (single-arm design)

BMI, body mass index; KL, Kellgren–Lawrence; mITT, modified intention-to-treat; MCII, minimal clinically important improvement; PP, per-protocol; SH, sodium hyaluronate; VAS, visual analog scale; WOMAC, Western Ontario and McMaster Universities Osteoarthritis Index.

Table S2 – Baseline demographics and clinical characteristics of the PP population.

**Characteristics**		**1% SH Trueba Davalillo study (*N*=89)**	**2% SH SOYA Trial (*N*=74)**	**p-value**
Age, median ± (Q1–Q3) (years)		61.0 (59.0 – 65.0)	69.0 (63.0 – 74.3)	< 0.001 [1]
Age, n (%)	< 65 years	66 (74.2)	25 (33.8)	< 0.001 [2]
	≥ 65 years	23 (25.8)	49 (66.2)	
Sex, n (%)	Male	34 (38.2)	18 (24.3)	0.065 [2]
	Female	55 (61.8)	56 (75.7)	
BMI median ± (Q1–Q3) (kg/m²)		28.6 (24.8 – 32.2)	27.2 (25.6 – 29.4)	0.021 [1]
BMI category, n (%)	Underweight: BMI < 18.5 kg/m²	0 (0.0)	1 (1.4)	< 0.001 [2]
	Normal: BMI 18.5 - 24.99 kg/m²	23 (25.8)	11 (14.9)	
	Overweight: BMI 25 - 29.99 kg/m²	30 (33.7)	62 (83.8)	
	Obese: BMI ≥ 30 kg/m²	36 (40.4)	0 (0.0)	
Painful side, n (%)	Bilateral	11 (12.4)	0 (0.0)	0.002 [2]
	Right	40 (44.9)	42 (56.8)	
	Left	38 (42.7)	32 (43.2)	
K&L Scale, n (%)	II	58 (65.2)	30 (40.5)	0.003 [2]
	III	31 (34.8)	44 (59.5)	
Paracetamol consumption, n (%)	No	25 (28.1)	42 (56.8)	< 0.001 [2]
	Yes	64 (71.9)	32 (43.2)	

BMI: body mass index; K&L: Kellgren-Lawrence; SH: sodium hyaluronate. [1]: p-value analyzed using the Mann-Whitney test. [2]: p-value analyzed using Fisher's exact test.

Table S3 – Relative change in pain intensity from baseline to 12 months in the PP population.

**Measure (%)**	**1% SH Trueba Davalillo study (*N* = 89)**	**2% SH SOYA Trial (*N*=74)**
Mean	33.53	40.81
Standard deviation	11.66	46.32
Standard error	1.23	5.38
Mean difference	7.28	
Standard error of the difference	5.52	
95% confidence interval	-3.71 – 18.27	

SH: sodium hyaluronate.

Figure S1 - Pain intensity scores (measured by the VAS) in the PP population. (A) Median pain intensity scores from baseline to 12 months of follow-up. (B) Relative change (%) in pain intensity scores from baseline at 6 and 12 months. *p*-values were calculated using the Mann–Whitney U test. SH: sodium hyaluronate; VAS: visual analog scale.

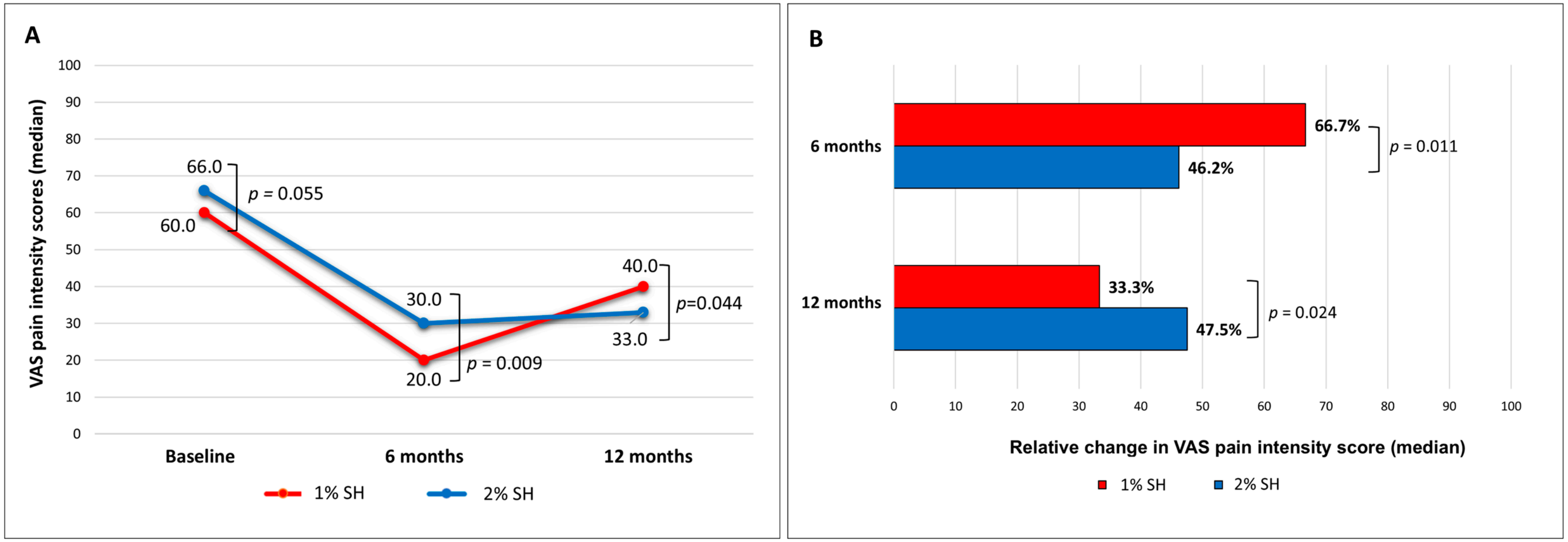

Table S4 – Multivariate analysis (ANCOVA) of the relative change in pain intensity (measured by VAS) and WOMAC scores at 12 months in the PP population.

**Outcome**	**Adjusted Mean Relative Change (95% CI)**<br>1% SH<br>Trueba Davalillo study<br>(*N*=89)	**Adjusted Mean Relative change (95% CI)**<br>2% SH<br>SOYA Trial<br>(*N*=74)	***p*-value**
Pain intensity (measured by VAS) adjusted for baseline pain scores	34.17 (27.43 – 40.91)	40.05 (32.65 – 47.45)	0.250
Pain intensity (measured by VAS) adjusted for baseline variables	34.26 (27.10 – 41.41)	39.94 (31.97 – 47.92)	0.336
Total WOMAC scores adjusted for baseline scores	41.93 (33.30 – 50.56)	41.08 (31.29 – 50.88)	0.913
Total WOMAC scores adjusted for baseline variables	42.31 (33.44 – 51.17)	40.63 (30.49 – 50.78)	0.839
WOMAC pain subscale scores adjusted for baseline scores	32.19 (23.43 – 40.94)	47.55 (37.69 – 57.405)	0.044
WOMAC pain subscale adjusted for baseline variables	33.21 (24.26 – 42.17)	46.31 (36.13 – 56.49)	0.106
WOMAC physical function subscale scores adjusted for baseline scores	44.48 (35.94 – 53.01)	38.94 (29.29 – 48.60)	0.463
WOMAC physical function subscale adjusted for baseline variables	44.73 (35.87 – 53.60)	38.63 (28.52 – 48.74)	0.453

VAS: visual analog scale; WOMAC: Western Ontario and McMaster Universities Osteoarthritis Index; CI: confidence interval; SH: sodium hyaluronate. *p*-values were calculated using the Mann–Whitney U test.

Figure S2 - Median WOMAC scores (A, C, E) and relative change from baseline (B, D, F) for total score (A–B), pain subscale (C–D), and physical function subscale (E–F) in the PP population. *p*-values were calculated using the Mann–Whitney U test. WOMAC: Western Ontario and McMaster Universities Osteoarthritis Index; SH: sodium hyaluronate.

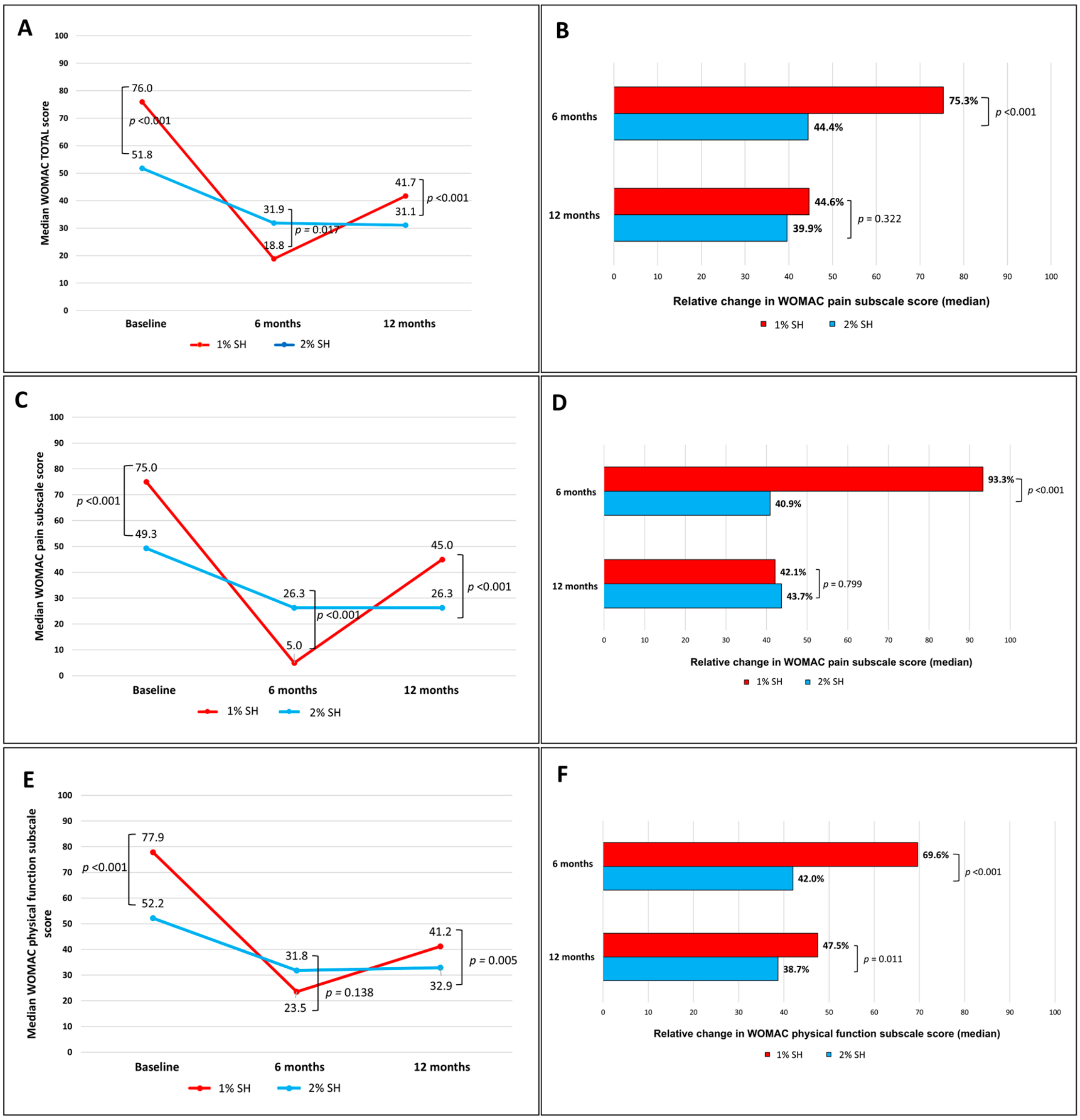

Figure S3 - Proportion of the PP population achieving the minimal clinically important improvement (MCII) for pain intensity (A) and WOMAC physical function (B) at 6 and 12 months. The MCII was defined as the proportion of patients achieving an absolute improvement of ≥15 mm from baseline. *p*-values were calculated using the Fisher's exact test. SH: sodium hyaluronate.

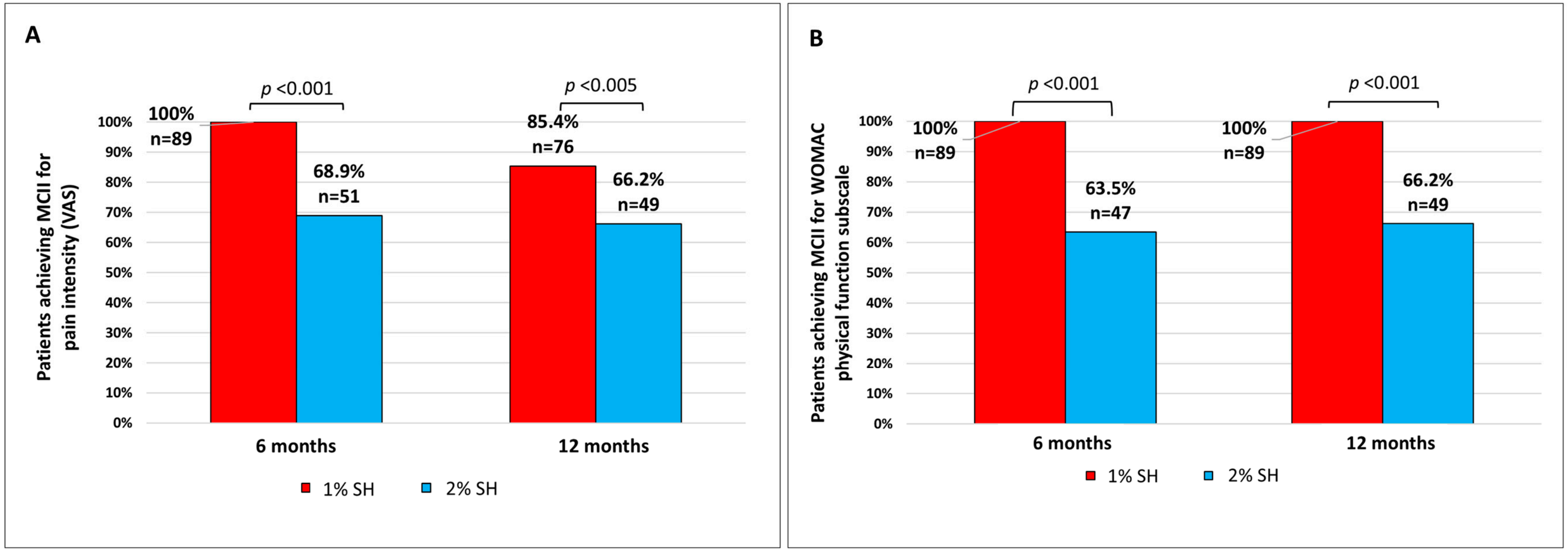

Table S5 – Post hoc, exploratory comparison of relative change in VAS pain and WOMAC physical function scores at 12 months in the PP population treated with 2% SH (SOYA trial) or betamethasone (Trueba Davalillo study).

Outcome	Group	Adjusted Mean Relative Change (95% CI)	SD	Min	Max	*N*	*p*-value
Pain intensity (measured by VAS)	Betamethasone (Trueba Davalillo study)	6.8 (4.1–9.4)	12.9	-50.0	33.3	91	<0.001
	2% SH (SOYA trial)	40.8 (30.1–51.5)	46.3	-90.2	100.0	74	
WOMAC physical function	Betamethasone (Trueba Davalillo study)	11.8 (10.4–13.3)	6.9	-2.7	45.8	91	<0.001
	2% SH (SOYA trial)	35.9 (24.4–47.5)	49.9	-199.7	99.7	74	

VAS: visual analog scale; WOMAC: Western Ontario and McMaster Universities Osteoarthritis Index; SD: standard deviation; CI: confidence interval; SH: sodium hyaluronate; Min: minimum; Max: maximum. *p*-values were calculated using the Mann–Whitney U test.
